# Pronóstico de caninos impactados según su posición en el maxilar superior mediante diferentes análisis radiográficos

**DOI:** 10.21142/2523-2754-1001-2022-096

**Published:** 2022-03-30

**Authors:** Clarisse Díaz-Reissner, Elke Pistilli, Rodrigo Caje, Clara Maldonado, Elena Jolay, Marta Ferreira-Gaona

**Affiliations:** 1 Direccion de Investigacion. Facultad de Odontologia. Universidad Nacional de Asuncion, Paraguay. cdiazr@odo.una.py, Universidad Nacional de Asunción Direccion de Investigacion Facultad de Odontologia Universidad Nacional de Asuncion Paraguay cdiazr@odo.una.py; 2 Instituto Latinoamericano de Estudios Superiores. Asuncion, Paraguay. elke.pistilli@gmail.com, rodrigocaje2@gmail.com, draclaritamr@gmail.com, elena.jolay@gmail.com Instituto Latinoamericano de Estudios Superiores Asuncion Paraguay elke.pistilli@gmail.com rodrigocaje2@gmail.com draclaritamr@gmail.com elena.jolay@gmail.com; 3 Direccion de Investigacion. Facultad de Odontologia. Universidad Nacional de Concepcion, Paraguay. martaf.baez@gmail.com Universidad Nacional de Concepción Direccion de Investigacion Facultad de Odontologia Universidad Nacional de Concepcion Paraguay martaf.baez@gmail.com

**Keywords:** diente canino, diente impactado, radiografía panorámica, cuspid, tooth, impacted, radiography, panoramic

## Abstract

**Introducción:**

La impactación de los caninos es una anomalía en la que el diente se halla impedido para erupcionar. Se produce cuando la erupción ha demorado y existe evidencia radiográfica.

**Objetivo:**

Determinar el pronóstico de caninos impactados según su posición en el maxilar mediante diferentes análisis: Ericson y Kurol, Warford y, Power y Short; en pacientes que acuden a consultorios privados en la ciudad de Asunción, entre los años 2015 y 2020.

**Metodología:**

El diseño es observacional descriptivo de corte transversal, mediante mediciones en radiografías panorámicas.

**Resultados:**

La muestra fue de 37 pacientes y de 48 caninos. Se encontró que la presencia de caninos impactados fue más frecuente en mujeres (69,44%), con ubicación unilateral (70,27%) y posición palatina (68,75%). En más de la mitad de los casos los pronósticos fueron desfavorables; sin embargo, la concordancia fue del 75%. Hubo mayor concordancia en el pronóstico de Warford con el de Power y Short (95,91%), mientras que la concordancia fue menor con el de Ericson y Kurol (81,25%).

**Conclusión:**

La frecuencia de caninos impactados en una muestra paraguaya fue mayor en mujeres, y están ubicados unilateralmente y posicionados por palatino. Es desfavorable en más de la mitad de los casos. Se sugiere combinar los análisis para emitir pronósticos.

## Abstract (en)

**Introduction:**

Canine impaction is an anomaly in which the tooth is prevented from erupting. It occurs when eruption has been delayed and there is radiographic evidence.

**Objective:**

To determine the prognosis of impacted canines according to their position in the maxilla by means of different analyses: Ericson & Kurol, Warford and Power & Short; in patients attending private clinics in the city of Asuncion from 2015 to 2020.

**Methodology:**

The design is observational descriptive, cross-sectional, using panoramic radiograph measurements. Results: The sample consisted of 37 patients and the 48 canines. It was found that the presence of impacted canines was more frequent in women (69.44%), with unilateral location (70.27%) and palatal position (68.75%). In more than half of the cases the prognosis was unfavorable, however, the concordance was only 75%. There was greater concordance in Warford's prognosis with that of Power & Short (95.91%), while the concordance was lower with that of Ericson & Kurol (81.25%).

**Conclusion:**

The frequency of impacted canines in a Paraguayan sample was higher in females, unilaterally located and positioned by palatine. Being unfavorable in more than half of the cases. It is suggested to combine the analyses for projection.

## INTRODUCCIÓN

Cuando se supera el tiempo para la erupción según la secuencia de cronología y el diente se encuentra aún dentro de hueso, se considera al diente impactado. Generalmente, los terceros molares son los dientes que más presentan este problema, seguidos de los caninos maxilares, siendo estos últimos más prevalentes por palatino que vestibular ^1^, aunque se han encontrado caninos en posiciones atípicas: invertido en la apófisis frontal del maxilar ^2^. 

En un estudio efectuado en China se ha encontrado que la impactación vestibular del canino se encuentra relacionada con una deficiencia anterior transversal que pueden ser tanto de origen dental como esqueletal, mientras que la impactación por palatino se relaciona con incisivos laterales pequeños o ausentes ^3^. Inclusive, se ha relacionado la impactación del canino por palatino con la profundidad de la silla turca del esfenoides ^4^. Sin embargo, en brasileños no se ha encontrado relación con el sexo, tipo de maloclusión, biotipo facial, agenesia y alteración del tamaño del incisivo lateral permanente ^5^.

En un estudio realizado acerca de los factores que afectan la severidad en los dientes retenidos, se concluyó que el ángulo de un diente impactado puede aumentar con la edad y que las mujeres suelen tener una impactación más severa que los hombres, especialmente en caninos maxilares. Por este motivo, es importante un tratamiento temprano y oportuno, y son relevantes inclusive los procedimientos de ortodoncia interceptiva ^6^. Por otra parte, en un metaanálisis, concluyeron que la localización y el ángulo, esto es, la inclinación mesial y horizontal inicial, son útiles para predecir la erupción espontánea en posición palatina de caninos maxilares permanentes ^7^.

Se debe tener en cuenta que la presencia de caninos impactados no genera síntomas, pero puede dejar secuelas como malposición dentaria palatina o vestibular, desplazamiento de las piezas dentarias, disminución de longitud del arco dental, reabsorción radicular interna o externa, formación de quistes odontogénicos, infección o dolor dentario ^8^.

Las radiografías confirman la presencia de un canino retenido, permiten la evaluación, la presencia y tamaño del folículo, así como la integridad corono-radicular, con lo cual relacionan al canino con los dientes adyacentes y su localización en sentido mesiodistal o vertical ^1^.

Generalmente, según la cronología de erupción, los caninos superiores son los penúltimos dientes permanentes en aparecer en boca, por eso es importante que erupcionen en sus alvéolos, debido a su importancia funcional, estética y dentaria ^9^. En las maloclusiones que involucran a los caninos retenidos, por lo general, se requiere un tratamiento de ortodoncia. Sin embargo, la corrección ortodóncica de los caninos retenidos con un patrón de erupción ectópico es un factor de riesgo para producir reabsorción apical de los dientes anteriores ^8^. En ese caso, la cirugía es necesaria, se hace una apertura hasta la corona del canino con reposición apical del colgajo cuando se encuentra por vestibular o simplemente liberando la corona del hueso y mucosa cuando está por palatino, respetando siempre la unión cemento-esmalte ^10^.

Se planteó como objetivo del estudio determinar los valores de medidas lineales horizontales de la arcada en pacientes atendidos en clínicas privadas entre los años 2015 y 2020. La importancia de la investigación de las distancias intercaninas e intermolares radica en que podríamos intervenir precozmente y lograr un tratamiento exitoso, y así evitar que las anomalías se instalen.

La detección temprana de caninos impactados es importante, especialmente cuando la pérdida prematura de los caninos deciduos ocasiona la falta de espacio para la erupción normal de los caninos permanentes; por tanto, el examen por imágenes resulta fundamental para diagnosticar y plantear el tratamiento oportuno según el pronóstico del caso.

## MATERIALES Y MÉTODOS

El diseño del estudio fue observacional descriptivo de corte transversal. Participaron radiografías de pacientes con caninos impactados superiores que acudieron a consultorios privados de la ciudad de Asunción entre los años 2015 y 2020. Fueron incluidas las radiografías panorámicas con calidad de imagen, que permitieron observar claramente con el negatoscopio las estructuras anatómicas requeridas para este estudio. Fueron excluidas las radiografías con presencia de fracturas de los maxilares, caninos ausentes u otra patología que imposibilitaron analizar adecuadamente el canino impactado. 

Se realizó la calibración de un examinador realizando los calcos de radiografías y análisis en pacientes que no formaron parte del estudio, con un examinador de referencia y experiencia. Se colocó la radiografía sobre el negatoscopio y una regla milimétrica en el extremo derecho, lo que permitió ampliar e imprimir la imagen a escala. Se solicitó dicho procedimiento a cada odontólogo para la remisión de radiografías.

**Figura 1:**
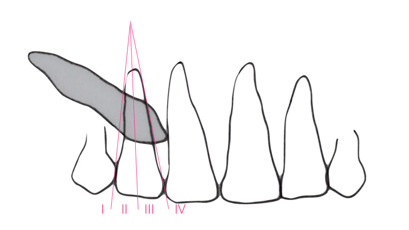
Análisis de Ericson y Kurol (modificación de Lindauer *et al*.)

**Figura 2:**
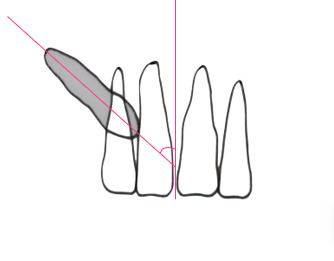
Análisis de Power y Short

**Figura 3:**
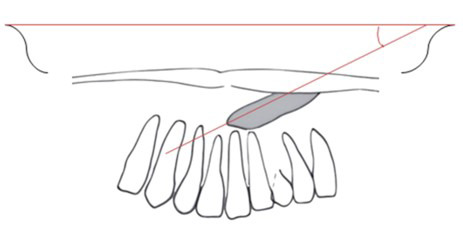
Análisis de Warford *et al*.

Se midió la posición de la corona según diferentes análisis radiográficos: a) Análisis de Ericson y Kurol (modificación de Lindauer *et al*.), que utiliza como referencia el incisivo lateral para trazar líneas que pasan por el centro del diente, por mesial y distal, y lo dividen en cuatro sectores. Se considera la ubicación de la cúspide del canino; es favorable hacia el sector I, que se encuentra hacia distal y desfavorable el sector IV hacia mesial (Figura 1), con un riesgo de impactación del 87% en el sector III y del 99% en el sector IV ^(11-13^). b) Análisis de Power y Short, por el cual se forma con un ángulo conformado por el eje longitudinal del canino y una línea media que pasa la espina nasal anterior (Figura 2). A medida que aumenta el ángulo, si supera los 31º, el pronóstico es desfavorable. c) Análisis de Warford *et al*., que utiliza también como referencia el eje longitudinal del canino que forma un ángulo con la línea bicondilar (Figura 3). Cuando supera los 75º, el pronóstico es favorable, y cuando es menor a 59°, es desfavorable ^14^.

Además, se midieron la posición en la arcada (palatina, media o vestibular, y unilateral o bilateral) y el sexo (masculino o femenino) registrado en la radiografía. El muestreo fue por conveniencia.

El protocolo fue aprobado por el Comité de Ética en Investigación de la Facultad de Odontología de la Universidad Nacional de Asunción Informe N.^o^ 22/22. Se solicitó permiso para acceder a los expedientes clínicos de los pacientes. Los datos de los pacientes fueron tratados de manera confidencial y solo fueron utilizados para los fines del estudio, y se presentaron los resultados de manera global. Mediante llamado telefónico, se solicitó a los colegas radiografías de pacientes que podian formar parte del estudio y cuyos datos fueron ser remitidos con el fin de la investigación. 

Se utilizó estadística descriptiva para presentar los resultados. Las variables cuantitativas se presentaron como porcentaje en gráficos. Se utilizó el programa Microsoft Excel 2019 para la carga de base de datos y su análisis.

## RESULTADOS

Formaron parte del estudio 37 pacientes, de los cuales 11 tenían caninos impactados bilaterales superiores, 12 en el lado derecho y 14 en el lado izquierdo. Entonces, en el 70,27% de los pacientes fue unilateral y en el 29,73%, bilateral. El 69,44% fueron mujeres y el 30,56%, varones. 

Por tanto, la muestra quedó conformada por 48 caninos impactados. Se encontraron por palatino el 68,75% de los caninos impactados y el 31,25%, por vestibular.

**Figura 4:**
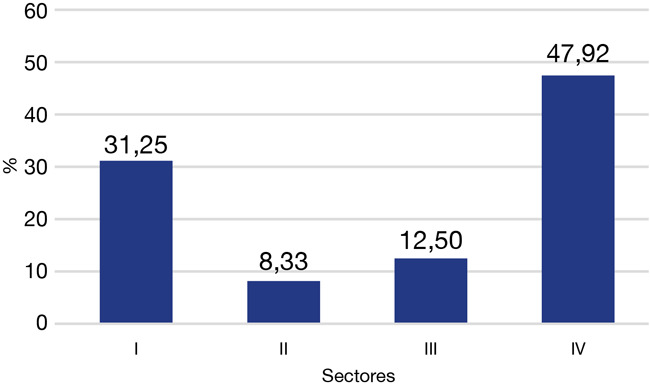
Distribución de caninos impactados por sector según el análisis de Ericson y Kurol con modificación de Lindauer *et al*. (n = 48)

Considerando que el sector I tiene pronóstico favorable y el sector IV desfavorable, se observa que el 47,92% tiene un pronóstico desfavorable, en los sectores III y IV (Figura 4).

**Figura 5:**
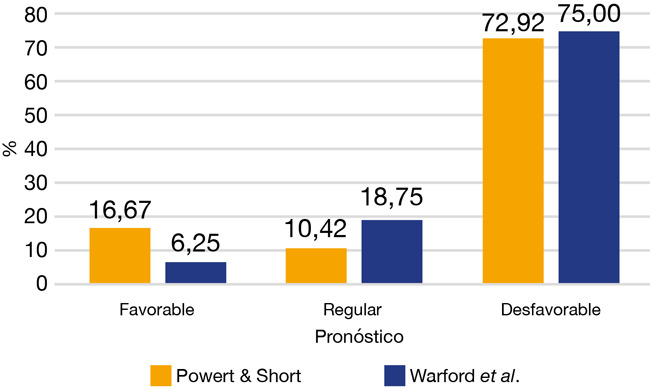
Distribución de caninos retenidos según análisis de Power y Short y Warford *et al*. (n = 48)

El 72,92% presentó un pronóstico desfavorable según el análisis de Power y Short y el 75,00% presentó un pronóstico desfavorable para el análisis de Warford *et al*. (Figura 5).

Al comparar el pronóstico con los tres métodos de análisis se encontró una concordancia del 75% entre ellos. Al comparar por análisis, se encontró que Waford con Power y Short coinciden en un 95,91% (46/48) de los pronósticos, mientras que en estos dos análisis, al compararlos con el análisis de Ericson y Kurol (modificación de Lindauer *et al*.), la concordancia fue del 81,25% (39/48).

## DISCUSIÓN

En este estudio se planteó como objetivo determinar el pronóstico que presentan los caninos impactados en el maxilar superior utilizando diferentes análisis radiográficos: Ericson y Kurol (modificación de Lindauer *et al*.), Warford y, Power y Short en pacientes que acuden a consultorios privados en la ciudad de Asunción de los años 2015 al 2020, cuyos análisis arrojaron como resultado un pronóstico desfavorable en el 60,42%, 72,92% y 75,00% de los casos, respectivamente.

Los caninos se encuentran en segundo lugar entre los dientes impactados con mayor frecuencia, seguidos de los terceros molares. Son del 1 a 3% en la población general ^15^. Sin embargo, en un estudio realizado en Serbia, los caninos impactados fueron los más prevalentes, con un 34,8% de anomalías dentales, y la más frecuente fue la impactación, especialmente del canino maxilar ^16^. El resultado es similar al de un estudio realizado en Italia, en el que la anomalía más frecuente fue el desplazamiento de caninos en un 7,5%, en una muestra de 5000 sujetos ^17^. En otro estudio encontraron que la forma del arco maxilar era más estrecha y más larga en el grupo canino impactado palatalmente, en comparación con el grupo canino impactado bucalmente, y el grupo canino impactado palatino tenía una bóveda palatina más profunda que el grupo canino impactado bucalmente ^18^. Por otro lado, se sugiere que un mayor grado de desplazamiento canino podría estar asociado con la reabsorción severa de la raíz incisiva ^19^ y que el tratamiento de los caninos impactados es más difícil en pacientes con prognatismo maxilar ^20^. También se menciona entre las causas principales la oligodoncia y las alteraciones de forma y tamaño del incisivo lateral, y el tratamiento elegido es la extracción del canino temporal siempre que las condiciones de espacio estén presentes ^21^.

Al comparar la frecuencia encontrada por sexo, el doble de los casos fueron mujeres, lo que coincide con otros estudios, como el realizado en Kosovo, en el que el 64% fueron en mujeres ^15^; en Serbia, en el cual fue significativamente mayor en mujeres ^16^, y también en adolescentes colombianos ^22^. Pero esto podría deberse en nuestro medio a que las mujeres acuden más al odontólogo, tienen tendencia a realizarse estudios y el tratamiento correspondiente.

En cuanto al lado de afectación, tuvieron similar distribución para bilaterales, tanto derecho como izquierdo; sin embargo, en Colombia fue más afectado el lado derecho ^22^, prevaleció la afectación unilateral en similar proporción al estudio en Kosovo donde fue del 75% ^15^ y en Colombia donde fue también unilateral en un 69,4% ^22^. Con nuestro estudio se podría pensar que, como la toma de muestra fue al azar, la distribución se mostró homogénea en cuanto al lado de afectación. 

Acerca de la ubicación, el doble se encontró la posición palatina, lo que difiere de un estudio realizado en adolescentes colombianos, pero coincide con otros estudios, como el realizado en Kosovo donde el 72,4% se encontró por palatino ^15^ y el 69,4% se encontró por vestibular en Colombia ^22^.

En caso clínico realizado en un adolescente de 12 años que se sometió a una tracción del canino izquierdo para una erupción guiada se obtuvo como resultado una oclusión funcional, mejoró la estética y se mantuvo estable luego de 1,5 años de seguimiento ^23^. Su presencia resulta importante en la eficacia masticatoria, dado que da estabilidad a la arcada dentaria, mantiene la estética y una expresión facial armoniosa. Es por esto que su detección temprana para el tratamiento de erupción guiada es de suma importancia ^24^.

En cuanto al pronóstico, en nuestro estudio, el 60,42% se encontró en los sectores III y IV, lo que es considerado desfavorable, y es casi el doble de lo reportado en Colombia, con el 35% en los sectores III y IV ^22^; pero estuvo más cerca al 48,5% reportado en el estudio de Warford *et al*. ^14^, quien estableció que la punta del canino es el predictor más importante para determinar la impactación del canino.

Acerca de la falta de concordancia en nuestro estudio entre los tres métodos de medición que fue del 75%, podría deberse que los análisis que utilizan medidas angulares como Warford *et al*. y Power y Short coincidieron entre ellos pero difirieron del análisis sectorizado de Ericson y Kurol con modificación de Lindauer *et al*., debido a que constituyen diferentes tipos de mediciones; resultado que coincide con el estudio de Upegui Zea *et al*. ^22^, quienes concluyeron que hubo asociación y concordancia fuerte y positiva entre los análisis de Power y Short y, Warford *et al*., pero no así con el de Lindauer *et al*. De acuerdo con esto, es válido realizar tanto el análisis angular como sectorial, de preferencia entre los angulares el de Power y Short porque consideran que evita sesgos relacionados con la ubicación del plano condilar utilizado como referencia en el análisis de Warford, ya que este utiliza la línea media como referencia, lo que resulta más sencillo de ubicar correctamente en la radiografía.

Como limitación del estudio se puede mencionar la falta de estimación de prevalencia de caninos impactados; la falta de seguimiento del paciente, que no permite corroborar la dificultad estimada; y el tamaño de muestra, que no resulta extrapolable.

Se recomienda realizar un estudio para conocer la prevalencia a nivel nacional, dado que no existen estudios que pudieran ser referentes. Así mismo, es necesario realizar un seguimiento para evaluar si concuerda la dificultad pronosticada con la encontrada en la clínica, lo que podrá permitir incluso poder validar el instrumento.

## CONCLUSIONES

Se encontró que la presencia de caninos superiores impactados fue más frecuente en mujeres, con ubicación unilateral y posición palatina. Los pronósticos desfavorables se dieron en más de la mitad de los casos, para los tres análisis; sin embargo, la concordancia fue del 75%.

Hubo mayor concordancia en el pronóstico entre el análisis de Warford y el análisis de Power y Short, mientras que la concordancia fue menor con el análisis de Ericson y Kurol con modificación de Lindauer *et al*. Debido a que los dos primeros son medidas angulares y el última sectorial, se sugiere complementar ambas medidas.

## Fuente de financiamiento:

Autofinanciado
